# Machine Learning and Wearable Sensors for the Early Detection of Balance Disorders in Parkinson’s Disease

**DOI:** 10.3390/s22249903

**Published:** 2022-12-16

**Authors:** Francesco Castelli Gattinara Di Zubiena, Greta Menna, Ilaria Mileti, Alessandro Zampogna, Francesco Asci, Marco Paoloni, Antonio Suppa, Zaccaria Del Prete, Eduardo Palermo

**Affiliations:** 1 Department of Mechanical and Aerospace Engineering, Sapienza University of Rome, 00184 Rome, Italy; 2 Mechanical Measurements and Microelectronics (M3Lab) Laboratory, Engineering Department, University Niccolò Cusano, 00166 Rome, Italy; 3 Department of Human Neurosciences, Sapienza University of Rome, 00185 Rome, Italy; 4 IRCCS Neuromed Institute, 86077 Pozzilli, Italy; 5 Department of Anatomical and Histological Sciences, Legal Medicine and Orthopedics, Sapienza University of Rome, 00185 Rome, Italy

**Keywords:** Parkinson’s disease, postural instability, machine learning, posturography, wearable sensors

## Abstract

Dynamic posturography combined with wearable sensors has high sensitivity in recognizing subclinical balance abnormalities in patients with Parkinson’s disease (PD). However, this approach is burdened by a high analytical load for motion analysis, potentially limiting a routine application in clinical practice. In this study, we used machine learning to distinguish PD patients from controls, as well as patients under and not under dopaminergic therapy (i.e., ON and OFF states), based on kinematic measures recorded during dynamic posturography through portable sensors. We compared 52 different classifiers derived from Decision Tree, K-Nearest Neighbor, Support Vector Machine and Artificial Neural Network with different kernel functions to automatically analyze reactive postural responses to yaw perturbations recorded through IMUs in 20 PD patients and 15 healthy subjects. To identify the most efficient machine learning algorithm, we applied three threshold-based selection criteria (i.e., accuracy, recall and precision) and one evaluation criterion (i.e., goodness index). Twenty-one out of 52 classifiers passed the three selection criteria based on a threshold of 80%. Among these, only nine classifiers were considered “optimum” in distinguishing PD patients from healthy subjects according to a goodness index ≤ 0.25. The Fine K-Nearest Neighbor was the best-performing algorithm in the automatic classification of PD patients and healthy subjects, irrespective of therapeutic condition. By contrast, none of the classifiers passed the three threshold-based selection criteria in the comparison of patients in ON and OFF states. Overall, machine learning is a suitable solution for the early identification of balance disorders in PD through the automatic analysis of kinematic data from dynamic posturography.

## 1. Introduction

Parkinson’s disease (PD) is the second most common neurodegenerative disorder worldwide [1]. It is primarily characterized by the death of the dopaminergic neurons in a specific region of the midbrain named substantia nigra pars compacta [2]. Indeed, the current therapeutic cornerstone in PD consists of replacement treatment with L-Dopa that significantly improves movements, although it is invariably associated with complications in the advanced stages of the disease (e.g., dyskinesia and fluctuations) [3]. Among parkinsonian motor symptoms, postural instability is one of the most debilitating [2,4]. Postural instability severely affects balance and causes frequent falls and injuries, leading to increased hospitalization, loss of independence and high mortality rates in PD [5]. The delayed recognition and controversial response to the dopaminergic therapy of postural instability is responsible for negative outcomes in patients with PD [5]. Accordingly, the early identification of postural instability is a primary concern in the clinical management of patients with PD in order to optimize therapeutic strategies, prevent injuries and support the individual’s autonomy. 

The current challenge resides in the low sensitivity of routine clinical examinations to early recognize postural instability in PD [6]. Indeed, clinical assessment usually detects postural instability only when the patient becomes symptomatic by complaining of balance issues and recurrent falls [7]. Dynamic posturography is an instrumental technique to objectively assess postural control under challenging conditions resembling free-living situations [8]. In 2021, Kamieniarz et al. [9] performed a study to detect balance changes in early stage of PD. They found that simple spatiotemporal parameters of the center of pressure (COP) were not enough to detect differences between PD and a healthy control (HC). Instead, power spectrum density (PSD) of the COP and sample entropy could detect early postural issues even if sample entropy failed to differentiate different states of the disease (Hoehn & Yahr H&Y-II and H&Y-III). In 2021, Yu et al. [10] also performed a quantitative analysis of postural instability in PD to detect early balance issues. They found that limits of stability in H&Y-I were not different from those of a HC, while they detected a pattern of decreases in postural stability during the progress of the disease. 

We have recently used dynamic posturography integrated with a network of wearable inertial sensors to examine postural control in a cohort of patients with PD without a clinically overt postural instability and without a history of falls [11]. Despite being clinically asymptomatic for postural instability, patients with PD showed abnormal reactive postural responses to axial rotations both under and not under L-dopa (i.e., ON and OFF state of therapy, respectively) [11]. Hence, our instrumental approach demonstrated a subclinical balance impairment in patients, pointing at the potential suitability of portable inertial sensors for the early recognition of postural instability in PD. 

The use of wearable sensors is part of the broader goal of integrating and interconnecting sensors and actuators with control systems and smart materials [12]. These kinds of sensors are becoming prominent in healthcare for measuring various biomechanical parameters [13,14]. Wearable sensors are easy to set up, relatively inexpensive and can be used in real-time since the processing phase is much shorter than the computing time required by some standard systems (e.g., marker tracking algorithms) [2]. However, the large-scale, high-dimensional data captured by wearable sensors requires sophisticated signal processing to transform it into scientifically and clinically valuable information, thus precluding their routine application in daily practice [15]. Providing clinicians with quantitative and automatic measures of motor and postural performance through portable sensors would allow the early detection and management of postural instability in PD. By automatically managing large volumes of data, machine learning algorithms have shown remarkable success in making accurate predictions for complex problems, including healthcare issues [16,17,18]. 

Another important role in the recent development of healthcare studies is played by machine learning solutions, which are often fed with data from various wearable sensors for the prediction of different patient health conditions [19,20,21,22]. In this scenario, a large number of researchers have already studied PD through inertial data and a machine learning approach, mostly involving gait. In 2018, Aich et al. [23] proposed a method to detect the freezing of gait (FoG). They asked 51 PD patients (36 with FoG episodes and 25 without FoG episodes) to perform gait tasks. They collected the inertial data from a 3D accelerometer on knees and extracted some features (such as average step time, stride time, step length, stride length, walking speed). They then trained four classifiers: Support Vector Machine (SVM), k-Nearest Neighbours (kNN), Decision Tree (DT) and Naïve Bayes. An accuracy of 89% was reported in the classification of FoG patients from no FoG patients with the SVM (rbf kernel). In 2015, Parisi et al. [24] presented a procedure to assess the severity of PD, automatically assigning UPDRS scores through a machine learning algorithm. They used portable sensors situated on the chest and both thighs and asked 24 PD patients to perform leg agility tasks and gait tasks. The best performance was that of kNN, with a maximum accuracy of 62%. In 2018, Caramia et al. [25] implemented a method to automatically divide PD patients from healthy subjects, examining the inertial data of 27 healthy controls and 27 PD patients: in particular, 8 subjects belonging to stage I, 9 to stage II and 8 to stage III on the Hoehn and Yahr (H&Y) scale. They were asked to perform a gait task. Different machine learning algorithms were used: Naïve Bayes, Linear Discriminant Analysis and Support Vector Machine. Average classification accuracy ranged between 63 and 75% among classifiers. In 2020, Aich et al. [26] realized a procedure to detect the ON/OFF state. Twenty idiopathic PD patients were asked to perform a gait task. Their inertial data were recorded by a 3D accelerometer on the knees. The extracted features were fed to a Support Vector Machine, k-Nearest Neighbors, Random Forest and Naïve Bayes. They found the best accuracy (96.7%) for the Random Forest classifier. The studies show no evidence of a machine learning algorithm outperforming others. Typology of the extracted features determined the most accurate algorithm in each case. 

Machine learning was also used for disease classification by considering other motor tasks. For example, in 2017 Jeon et al. [15] used machine learning for the assessment of the severity of PD in 85 PD patients. They collected inertial data from an accelerometer and a gyroscope situated on the wrist during a resting task and trained five types of classifiers: Decision Tree, Linear Discriminant Analysis, Random Forest (RF), k-Nearest Neighbors and Support Vector Machine. They found the best accuracy (85.5%) with the Decision Tree algorithm. In 2010, Cancela et al. [27] studied the automatic assessment of the severity of PD and the assignment of UPDRS score. They analyzed inertial data from IMUs situated on the limbs and the trunk of 20 idiopathic PD patients during daily life activity. They used four classifiers: Support Vector Machine, k-Nearest Neighbors, Decision Tree and Artificial Neural Network. The highest accuracy of 86% was achieved by SVM. 

The use of machine learning recently also appeared in posturography. Exley et al. [28] used it to predict UPDRS motor symptoms using force platforms during quiet standing. Features were obtained from quiet standing data while UPDRS-III subscores were the target variables. They compared seven machine learning algorithms for the classification: ridge and lasso logistic regression, Decision Tree, k-Nearest Neighbors, Random Forest, Support Vector Machine (rbf kernel) and Extreme Gradient Boosting. For body bradykinesia and hypokinesia, postural stability, rigidity and tremor at rest subscores, different algorithms performed the best in each case, but all of them had an accuracy lower than 80%.

In 2021, Fadil et al. [29] tested 19 PD and 13 HC. Patients were asked to lie supine on a tilt table for 5 min. The table was then tilted to 70 degrees for 15 min inducing an orthostatic challenge. Then, patients stood upright on a force platform and COP data were recorded. They used these data to feed six machine learning algorithms: Random Forest, Decision Tree, Support Vector Machine, k-Nearest Neighbors, Gaussian naïve Bayes and Neural Network (NN). Best performing algorithm was RF with an accuracy of 81%. They also found that PD patients were better differentiated from HC using time domain features compared to other feature groups. 

Most of the related literature studies are focused on the classification of PD gait disturbances in comparison to healthy subjects, neglecting postural instability, especially in the early stages of the disease. Moreover, despite the fact that studies for the automatic classification and scoring of PD severity using machine learning algorithms are copious, the classification of ON/OFF medication state still needs to be explored. 

Intending to fill this gap in the research, this study examines machine learning approaches to objectively evaluate early postural instability in PD, comparing reactive postural responses in patients with respect to healthy subjects when yaw rotations are pro-vided, trying to detect early asymptomatic postural abnormalities and verify whether those changes improve following dopaminergic therapy (i.e., OFF and ON state of therapy). Towards this aim, we analyzed and compared the results of four types of machine learning algorithms with different kernel functions.

## 2. Materials and Methods

### 2.1. Subjects

Twenty parkinsonian patients (1 woman and 19 men with a mean age of 67.7 ± 8.6 years) and a healthy control of fifteen subjects of similar age (mean age 65.2 ± 3.4 years) were enrolled in this study. The Department of Human Neurosciences of Sapienza University of Rome, Italy, enrolled PD patients according to specific inclusion criteria: diagnosis of idiopathic PD based on current criteria; no clinically evident postural instability; last year with no history of falls; score 1–2 on the Hoehn and Yahr scale; walk and upright stance independence; balance not affected by dementia (Mini-Mental State Examination-MMSE > 24), dyskinesia and comorbidities induced by L-Dopa.

An expert neurologist evaluated PD motor symptoms through the following clinical scales: Hoehn & Yahr scale and MDS-UPDRS (Movement Disorders Society-Unified Parkinson’s disease rating scale) part III. For the clinical assessment of balance, the postural instability/gait difficulty score (PIGD) and the Berg balance scale (BBS) were evaluated. 

PD patients were tested both on the ON and OFF medication states. The OFF state was evaluated at least 12 h after the usual dopaminergic treatment, while the ON state was assessed one hour after. For each patient, the L-Dopa Equivalent Daily Dose (LEDD) was calculated. Patients did not receive other neuropsychiatric medications possibly affecting balance at the time of the study. Written informed consent to the study, approved by the institutional review board following the Declaration of Helsinki, was given by all enrolled patients.

The number of subjects was chosen to guarantee an adequate dimensionality of the training, validation and testing datasets.

More information is reported in Table 1.

### 2.2. Experimental Setup

To perform the experiment, subjects stand on a robotic platform (RotoBit^1D^) in an upright position, with arms hanging vertically and feet externally rotated a preferred amount. The RotoBit^1D^ is a flat, rigid and round-shaped robotic platform with a polyethylene rotating disk with a height of 0.15 m and a diameter of 0.5 m, which allows a comfortable upright bipedal stance without narrowing feet. The RotoBit^1D^ is equipped with the following components: a SANYO DENKI servo motor (maximum torque = 1.96 Nm); a speed reducer; an incremental encoder; a toothed belt (PowerGrip HDT). This robotic platform has already been used in the study of various postural parameters [30,31] thanks to an ad-hoc LabVIEW software program (2014, National Instruments, Austin, TX, USA) that controls the robotic platform, providing sinusoidal rotation around the vertical (yaw) axis chosen for this study. 

Kinematics of axial body segments was gathered using three IMUs (MTw, Xsens Technologies-NL), each equipped with a 3-axes accelerometer, a 3-axes gyroscope and a 3-axes magnetometer. Wearable sensors were placed on the head, trunk and pelvis of participants through elastic belts to avoid relative movement between sensor and body. In more detail, the IMU of the head was placed under the supraciliary arc over the frontal bone, the IMU of the trunk was placed on the sternum body under the suprasternal notch and the IMU of the pelvis was placed just below the anterior sacral promontory and centered on the median sacral crests. Each subject was instrumented by the same expert operator to guarantee consistent sensor positioning on the body locations. IMUs data were acquired by MT Manager software, which let to visualization of inertial and magnetic quantity, detect pitch, yaw and roll angles, and understanding of 3D sensor orientation in real-time during the experimental session.

Both systems, the RotoBit^1D^ and the IMUs, were simultaneously triggered through the RotoBit^1D^ LabVIEW software. More specifically, an external trigger, i.e., a square signal ranging from 0 to +3 V, was provided to the IMUs both at the start and end of each trial.

**Figure 1:**
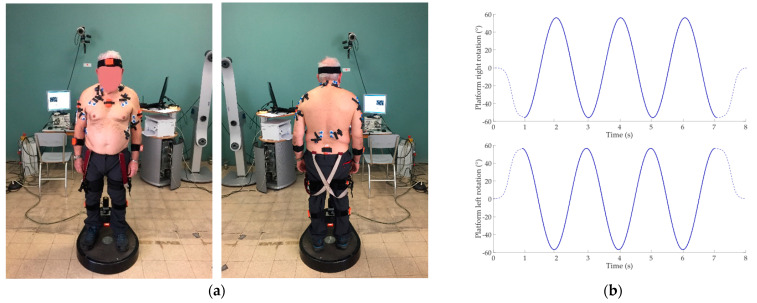
(**a**) Experimental setup and sensor placement on subject; (**b**) Right and left sinusoidal trajectories provided through the Rotobit^1D^ during the low perturbation condition. Dashed lines represent the sigmoidal trajectory of the beginning and the end of the acquisition. Continuous line represents the sinusoidal trajectory.

Experimental setup and trajectories of the sinusoidal perturbation are shown in Figure 1.

### 2.3. Experimental Protocol

Before each recording session, all subjects were asked to perform a functional calibration procedure (FC). The FC consists of standing and sitting tasks, each lasting 5 s and allow for overcoming incorrect misalignment between body and sensor orientation [32]. 

Subjects were asked to stand in their comfortable upright position on a robotic platform, facing a 2 m distant red cross placed in the wall in front of them. Feet were symmetrically placed over the center of the platform. Although all participants were instructed on the protocol before starting the experimental session, no instructions were provided on the postural strategies to be adopted to maintain balance. 

After performing the FC procedure, each subject was elicited though sinusoidal perturbation around the vertical axis. Three sinusoidal perturbations were provided: (i) Low Perturbation (LP), consisting of sinusoidal perturbation with a frequency of 0.2 Hz, peak amplitude of ±55° and a peak angular acceleration of 0.25°/s^2^; (ii) Medium Perturbation (MP), consisting of sinusoidal perturbation with a frequency of 0.3 Hz, peak amplitude of ±55° and a peak angular acceleration of 0.40°/s^2^; (iii) High Perturbation (HP), consisting of a sinusoidal perturbation with a frequency of 0.5 Hz, peak amplitude of ±35° and a peak angular acceleration of 0.50°/s^2^.

Each perturbation was repeated three times and starting both on the right and left side of the subject. Thus, a total of 18 trials were performed for each participant. Types of tasks are summarized in Table 2.

To make the subjects blind to the specific perturbation and avoid habituation or anticipatory strategies, perturbations were delivered randomly. To avoid bias due to similar task sequence, the order of the trials across the subject was randomized. Additionally, a sigmoidal wave was added at the start and at the end of the sinusoidal trajectory in order to avoid sudden variation in starting/stopping velocity.

### 2.4. Data Analysis and Features Extraction

Similar to [11], two types of features were extracted from the IMUs data: the range of motion of each body segment (head, trunk and pelvis) in AP and ML directions and the reciprocal body segment rotations, represented by the Gain Ratio (G) and the Phase Shift (φ).

To estimate the displacement of the head, trunk and pelvis in both the medio-lateral (ML) and antero-posterior (AP) directions, the acceleration signal of the inertial sensors was rotated in the global coordinate frame. Through the quaternion-derived rotation matrix, gravitational acceleration was removed. Then, the acceleration signal was straightforwardly integrated. Next, a zero-lag first-order Butterworth high-pass filter filtered the velocity with a cut-off frequency of 0.2 Hz for the AP and ML components and the body displacement was obtained through a second integration and filtering process. In this way, the range of motion of each body segment in the ML (ROM-ML) and AP (ROM-AP) directions were computed and expressed in mm. The reciprocal body segment rotations were also examined. The angular rotation of the pelvis, trunk and head around the longitudinal axis was computed according to previously reported procedures [33]. The Fast Fourier Transform was used to calculate the Gain Ratio (G) and the Phase Shift (φ) indices. In detail, the G index is calculated as the ratio of the maximum amplitudes of fundamental waves from distal and proximal body signals at the same frequency. The φ index was calculated in the Fourier domain as the difference between the phase angles of the two signal’s Fourier transform at the frequencies with the maximum amplitude. Values of G < 1 are associated with a lower amplitude of the yaw angle of the distal segment compared to the proximal one, whereas G > 1 are associated with a larger amplitude. A delay is connected to values of φ > 0, while an anticipation in the yaw phase between distal and proximal segments is related to values of φ < 0. Values that are close to 0 represent a perfect phase match the two segments.

In summary, each subject was asked to perform six different types of tests (see Table 2) and four features were extracted (ROM-ML, ROM-AP, G-relative and φ-relative) for each body segment (head, trunk and pelvis). Thus, the total number of the extracted features was 72, as combinations of 3 body segments × 6 tests × 4 extracted features.

### 2.5. Machine Learning: Training and Validation

Four types of machine learning algorithm were considered with different kernel functions: (i) Fine (DT_F_), Medium (DT_M_) and Coarse (DT_C_) Decision Tree; (ii) Fine (KNN_F_), Medium (KNN_M_), Coarse (KNN_CO_), Cosine (KNN_CS_), Cubic (KNN_CB_) and Weighted (KNN_W_) K-Nearest Neighbour; (iii) Linear (SVM_L_), Quadratic (SVM_Q_) and Cubic (SVM_CB_) Support Vector Machine; (iv) Artificial Neural Network (ANN). The neural network was a shallow ANN with 1 hidden layer with 10 neurons and 1 output layer with 2 neurons. 

To train the classifiers, 50-fold cross validation was used in DT, kNN and SVM and Leave One Subject Out Cross-Validation (LOSOCV) was used in ANN. Machine learning algorithms were implemented using the MATLAB (v.2020b, MathWorks, Natick, MA, USA) program.

These algorithms were chosen as they are some of the most commonly used and easy to implement.

The classification task consisted of four separate classification experiments:

1. PD vs. HC: which distinguishes between PD patients and healthy subjects;
2. OFF vs. HC: which distinguishes between patients in OFF state and healthy subjects;
3. ON vs. HC: which distinguishes between patients in ON state and healthy subjects;
4. OFF vs. ON: which distinguishes between patients in OFF state and patients in ON state.

In total, 52 machine learning classifiers were tested, as combinations of 13 types of machine learning algorithms × 4 classification experiments. 

### 2.6. Performance Evaluation

**Figure 2:**
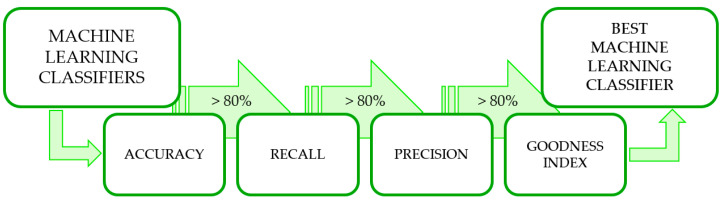
Flow chart for the identification of the best-performing classifiers.

To individuate the best performing machine learning algorithms among the 52 tested, confusion matrices were first extracted. Then, three threshold-based selection criteria [34] and one evaluation criteria were applied, similar to [35]. For all criteria, an 80% threshold was set (Figure 2).

The first selection criterion was based on accuracy, which represents the percentage of correct classification. It was calculated as:

(1) $$ACC=\left[\frac{TP+TN}{P+N}\right]\times 100$$

where *TP*, *TN*, *P* and *N* are the true positive, true negative, number of positive and negative, respectively. 

The second selection criterion was based on the recall or True Positive Rate (*TPR*), which represents the probability that an actual positive will test positive. It was calculated as:

(2) $$TPR=\left[\frac{TP}{TP+FN}\right]\times 100$$

(3) $$TNR=\left[\frac{TN}{TN+FP}\right]\times 100$$

where *TNR* stands for True Negative Rate, *FN* stands for false negative and *FP* stands for false positive.

The third selection criterion was based on the precision or *PPV*, which represents the percentage of true positive with respect to all positive. It was calculated as:

(4) $$PPV=\left[\frac{TP}{TP+FP}\right]\times 100$$

where *FP* is the number of false positive. 

According to the reported selection criteria, both type I and II errors were taken into account when evaluating the robustness of the classifier. The threshold applied was chosen to be 80% since it is a typical value derived from the literature for accepting a classifier as good [35]. 

The evaluation criterion was based on the Goodness index (*G*), which represents the Euclidean distance between the evaluated point in the ROC space and the point [0 1], representing the perfect classifier. It was calculated as:

(5) $$G=\sqrt{{\left(1-TPR\right)}^{2}+{\left(1-TNR\right)}^{2}}$$

where *TPR* and *TNR* are the true positive rate and true negative rate, respectively.

*G* has values included between 0 and √2. If *G* ≤ 0.25, a classifier can be considered as optimum, if 0.25 < *G* ≤ 0.70, it can be considered a good classifier, if *G* = 0.70 it can be considered a random classifier and if *G* > 0.70 it can be considered a bad classifier [35]. By analyzing *G* value results, the best-performing classifier could be evaluated.

**Figure 3:**
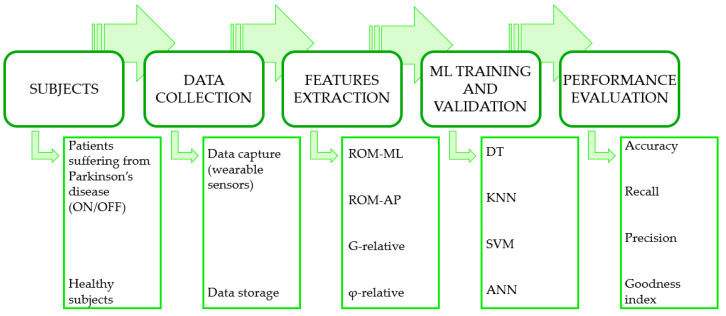
Complete process flow of the experiments.

Figure 3 summarizes the complete process flow of the experiments.

## 3. Results

The evaluation of the machine learning algorithms was carried out by considering accuracy, recall (or *TPR*) and precision (or *PPV*). The accuracy is used to evaluate the number of correct classifications, while the recall evaluates the number of data samples correctly identified in a particular class. Finally, the confidence to belong to a particular class for a particular prediction is defined by the precision. Furthermore, the goodness index was calculated and used to assess the best machine learning algorithm for each classifier and dataset combination.

### 3.1. First Selection Criterion

The accuracy of each classifier is reported in Table 3. To calculate *TN*, *TP* and accuracy, we defined the “positive” outcome for the different classifiers as follows: (i) for PD vs. HC “positive” is PD class; for OFF vs. HC, the OFF class; for ON vs. HC, the ON class; for OFF vs. ON, the ON class. 

Twenty-nine out of the 52 machine learning classifiers passed the first selection criterion and reached an accuracy equal to or higher than 80%. More specifically, the PD vs. HC classifier achieved a maximum accuracy of 95.6%. The OFF vs. HC classifier came up to a maximum accuracy of 90.9%. The ON vs. HC classifier reached a maximum accuracy of 89.9%. The OFF vs. ON classifier exhibited a top accuracy of 84.2%. It can be observed that the fine k-Nearest Neighbors algorithm showed the best performance in terms of accuracy for all classifiers, except for the OFF vs. HC. In this case, the best accuracy was reached using the coarse Decision Tree.

### 3.2. Second Selection Criterion

The *TPR* of the classifiers that met the first selection criterion are reported in Table 3. 

Twenty-eight out of the 29 classifiers passed the selection criterion based on the recall value. It can be noticed that the highest value of recall (100%) was reached by many algorithms among different classifiers.

### 3.3. Third Selection Criterion

Table 3 shows the *PPV* of the overall classification model that fulfilled the first and second selection criteria. 

Twenty-one out of 28 classifiers passed the third precision-based selection criterion, reaching a *PPV* at least of 80%. In detail, the PD vs. HC classifier achieved a *PPV* of 97.0%. The OFF vs. HC classifier came up to a *PPV* of 94.7%. The ON vs. HC classifier reached a *PPV* of 87.9%. In the OFF vs. ON classifier, no tested algorithms passed the precision threshold.

### 3.4. Goodness Index

The goodness index of all machine learning algorithms that passed the three selection criteria is exposed in Table 3. 

Twelve out of 21 could be catalogued as “good” classifiers because their G-index was 0.25 < *G* < 0.70. Nine out of 21 classifiers could be classified as “optimum” classifiers because their G-index was ≤ 0.25, such as DTm, kNNf, ANN for PD vs. HC; DTf, DTm, DTc, kNNf, SVMcu and ANN for OFF vs. HC; kNNf for ON vs. HC.

### 3.5. Best Performing Algorithms

The most important characteristics and parameters of the three best-performing algorithms, which present the lowest G-index for each classifier, are reported in Table 4. It can be noticed that the training time is short and the prediction speed is high.

**Figure 4:**
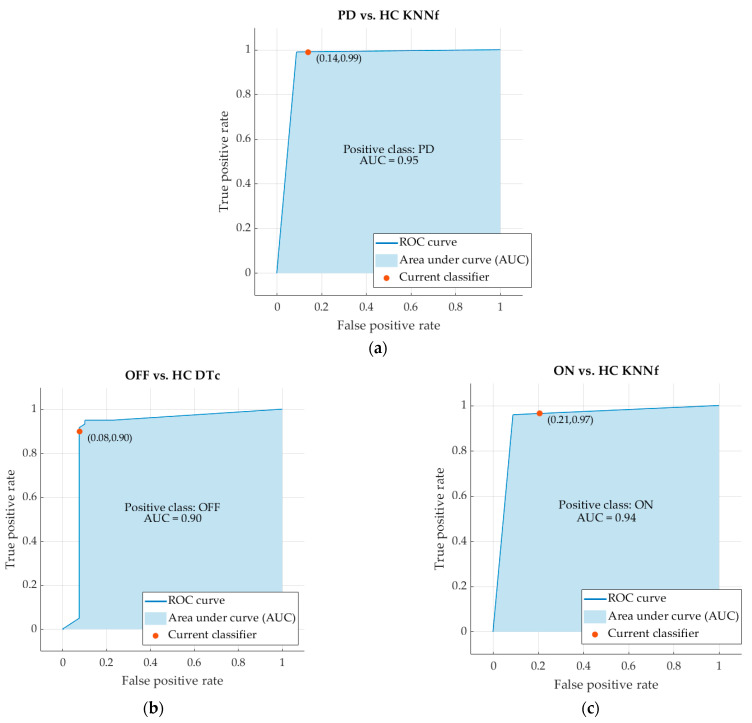
ROC curves and AUC values of the classifiers PD vs. HC (**a**), OFF vs. HC (**b**) and ON vs. HC (**c**).

The ROC curve and, in particular, the Area Under the Curve (AUC) are usually used for evaluating the accuracy of classifiers. The ROC curves and the AUC values of the best machine learning classifiers are reported in Figure 3. In more detail, the AUC of the classifier PD vs. HC is 0.95. The AUC of the classifier OFF vs. HC is 0.90. The AUC of the classifier ON vs. HC is 0.94 (Figure 4).

## 4. Discussion

In this study, we used and compared four types of machine learning algorithms, including Decision Tree (Fine, Medium, and Coarse), k-Nearest Neighbor (Fine, Medium, Coarse, Cosine, Cubic and Weighted), Support Vector Machine (Linear, Quadratic and Cubic) and Artificial Neural Network, to automatically classify patients with PD and healthy subjects based on reactive postural responses to yaw perturbations. Algorithms were fed with data obtained from portable inertial sensors placed on the body during the balance perturbation test. We have demonstrated that, when considering balance control, machine learning algorithms are able to accurately distinguish PD patients from healthy subjects, irrespectively of their state of therapy. By contrast, different therapeutic conditions are not efficiently identified in PD based on reactive postural responses, as demonstrated by the suboptimal performance of classifiers when comparing patients in ON and OFF state of therapy. 

The methodological approach adopted in this study allowed us to exclude a number of confounding factors possibly leading to the misinterpretation of findings. To specifically focus on asymptomatic and subclinical balance disorders, only PD patients without a history of falls and without clinically overt postural instability have been recruited. All patients have been carefully characterized clinically by means of standardized scales, investigating both motor and cognitive functions. Functional calibration procedures have been performed before all recording sessions to prevent the misalignment between subjects’ bodies and sensors orientation. Finally, experimental sessions have been conducted randomly according to the therapeutic state as well as the direction and frequency of postural perturbations to avoid possible carry-over effects. 

As a first finding, a relevant number of classifiers (i.e., 21 out of 52) successfully passed the three selection criteria (i.e., accuracy, recall and precision) provided for the selection of the best-performing algorithm by considering a threshold of 80%. Among these, most (i.e., 20 out of 21) were considered “good” classifiers based on their G-index. This result supports the hypothesis that subclinical changes of balance control occur in PD patients before postural instability becomes clinically evident, thus allowing the distinction from healthy subjects based on the analysis of reactive postural responses. Indeed, as already reported elsewhere [11], compared to healthy subjects, our cohort of patients showed increased displacement of axial body segments in the medio-lateral direction and reduced lumbo-sacral mobility during yaw perturbations, fully in line with a number of previous observations [36,37,38]. 

Only nine classifiers presented an “optimum” performance in distinguishing PD patients from healthy subjects according to their G-index. Among these, the kNNf was the most efficient classifier in the automatic detection of PD when comparing patients with healthy subjects, irrespective of their therapeutic state. Only the DTc showed a slightly better performance than the kNNf in the comparison between patients in OFF state and healthy subjects (i.e., G-index 0.12 vs. 0.18). Overall, the three best-performing algorithms (kNNf for PD vs. HC, DTc for OFF vs. HC and kNNf for ON vs. HC) showed high performance both in the training and prediction phases with a short training time and high prediction speed (Table 4). Indeed, the high accuracy values shown in Table 4 fully agree with the ROC-AUC values (between 0.90 and 0.97), in line with previous studies adopting a similar approach to assess other parkinsonian symptoms [15,39]. 

When looking at the performance of the best classifiers, an accuracy of 95.6% (kNNf), 90.9% (DTc) and 89.9% (kNNf) was reached in the distinction of healthy subjects from all patients, patients in OFF state and patients in ON state, respectively. These performances are similar or even better than those reported in previous studies adopting inertial data and machine learning to differentiate PD patients from healthy subjects based on specific motor symptoms (Table 5) [23,25,40,41,42].

It is likely that the mild differences in the classification performance between our and other studies mostly rely on the specific motor tasks adopted by various authors. Indeed, most previous studies primarily focused on gait abnormalities in PD patients, also including paroxysmal disorders such as FoG [23,25,40,41]. According to the high accuracy of our algorithms in classifying PD patients based on their balance control, our results expand previous findings by proposing reactive postural responses to yaw perturbations as alternative measures to recognize PD patients through machine learning algorithms. Moreover, our approach provides clinicians with a new sensitive tool to easily monitor postural abilities in PD patients over time and early recognize subclinical balance disorders. 

It can be seen that more complex deep learning techniques can lead to better results that allow discrimination of PD even in real-life situations [42].

Concerning the automatic classification of PD patients in ON and OFF states, the kNNf reached an accuracy of 84.2% and a *TPR* of 95% in distinguishing the different therapeutic conditions. However, owing to a *PPV* of 78.1%, the third selection criterion has failed and, consequently, all machine learning algorithms for the detection of the OFF and ON states presented a suboptimal performance. This finding is apparently in contrast with previous studies successfully using machine learning to distinguish PD patients under and not under L-Dopa [26,43,44,45]. However, it should be considered that balance disorders are usually refractory to dopaminergic therapy [7,37,46,47]. By contrast, previous studies successfully classifying ON and OFF states in PD considered motor symptoms that are largely responsive to L-Dopa, such as gait disorders [26,43,44,45,48]. Therefore, the suboptimal performance of our machine learning algorithms in distinguishing patients in ON and OFF states would reflect unchanged reactive postural responses after the L-Dopa intake, suggesting that, in PD, neurodegeneration involves neurotransmitter systems other than the dopaminergic one since the early stages of the disease [11]. Accordingly, these findings further support the potential usefulness of our approach to early recognize balance disorders and implement alternative therapeutic strategies, including tailored rehabilitative training, to prevent harmful injuries in PD.

The use of Rotobit complicates the reproducibility of the procedure, but any other source of controlled axial perturbation to the patients could be applied in a clinical setting. However, building on the results of this study, it would be possible in the future to identify a methodology in which the patient is placed on the perturbating platform with the three low-cost and easy-to-position wearable sensors. Using the best-performing machine learning (kNN), this will allow us to conduct an evaluation to add to the others clinical assessments of diagnosis of PD. Furthermore, to strengthen the identification of postural problems, the number of sensors applied could be increased when needed. Therefore, the use of a simple system and basic machine learning algorithms could be an excellent support to clinicians for the early identification of postural disorders due to Parkinson’s disease. 

## 5. Conclusions

This study has provided a new quantitative, reliable and useful tool based on dynamic posturography and machine learning to early recognize and objectively monitor balance control in patients with PD. The proposed data acquisition procedure is fast and simple, as is the equipment, which is composed of only three easy-to-place wearable inertial sensors. The inertial data can be stored, analyzed at a later time and used to train machine learning classifiers. This work points out how machine learning algorithms could successfully distinguish healthy subjects from PD patients without clinically overt postural instability, both in OFF and ON state, by analyzing reactive postural responses to yaw perturbations. Among the thirteen machine learning techniques used in this study, fine k-Nearest Neighbors outweighs all the other classifiers, detecting the two conditions (PD patients and healthy subjects) with a maximum accuracy of 95.6, recall of 99.0 and precision of 95.2%. Accordingly, this method may help to early recognize and address balance disorders in PD. In the future, a simulation model based on the data from this study could be developed to increase the possibility of validating the results. Furthermore, future developments foresee the possibility of applying this method to distinguish different stages of PD and monitor the disease progression, as well as to help in the differential diagnosis of atypical forms of parkinsonism, such as progressive supranuclear palsy and multiple system atrophy. 

## Figures and Tables

**Table 1:** Clinical features of patients with Parkinson’s disease.

Patients	PD Onset	H&Y	MDS-UPDRSIII	MMSE	PIGD	BERG
1	2017	1.5	27	30	1	56
2	2010	2	41	30	6	47
3	2013	1.5	26	30	4	50
4	2010	2	30	30	2	56
5	2013	1.5	15	30	2	55
6	2010	2	43	30	2	56
7	2015	1.5	24	30	1	56
8	2015	2	37	29	1	55
9	2011	2	45	25	7	45
10	2015	1.5	25	29	2	56
11	2016	1.5	22	28	1	55
12	2015	1.5	28	29	2	52
13	2018	1	14	29	2	56
14	2015	1.5	21	30	4	56
15	2015	1.5	21	25	2	50
16	2012	2	24	30	3	52
17	2010	2	25	30	5	54
18	2014	1	12	30	3	54
19	2016	1	10	30	2	52
20	2015	2	28	30	7	46
Mean	-	1.7	25.9	29.2	2.9	52.9
SD	-	0.5	9.8	1.5	1.9	3.6

**Table 2:** Resume of tasks provided by the Rotobit^1D^.

Perturbation	Low	Medium	High
Frequency (Hz)	0.2	0.3	0.5
Peak amplitude (°)	±55	±55	±35
Peak angular acceleration (°/s^2^)	0.25	0.40	0.50

**Table 3:** Percentage values of each classifier for each criterion: accuracy (*ACC*), which represents the percentage of correct classification; recall (*TPR*), which represents the probability that an actual positive will test positive (positives are PD in PD vs. HC, OFF in OFF vs. HC and ON in ON vs. HC and OFF vs. ON); precision (*PPV*), which represents the percentage of true positive with respect to all positive; goodness index (*G*), which represents the Euclidean distance between the evaluated point in the ROC space and the point [0 1], representing the perfect classifier. Values exceeding the threshold and passing to the next selection are shown in green, those not exceeding the threshold are shown in white and values not passed are shown with “/”.

		DTf	DTm	DTc	kNNf	kNNm	kNNc	kNNco	kNNcu	kNNw	SVMl	SVMq	SVMcu	ANN
PD vs. HC	*ACC*	84.7	84.7	86.9	95.6	86.1	73.7	86.1	83.9	89.1	88.3	89.1	92.7	88.3
	*TPR*	89.1	89.1	93.1	99.0	100	/	97.0	100	100	100	99.0	100	88.3
	*PPV*	90.0	90.0	89.5	95.2	84.2	/	86.0	82.1	87.1	86.3	87.7	91	97.0
	G	0.30	0.29	0.31	0.14	0.52	/	0.45	0.61	0.42	0.43	0.39	0.28	0.16
OFF vs. HC	*ACC*	88.9	88.9	90.9	89.9	79.8	60.6	77.8	75.8	83.8	79.8	83.8	88.9	82.8
	*TPR*	86.7	86.7	90.0	95.0	/	/	/	/	100	/	95.0	96.7	81.2
	*PPV*	94.5	94.5	94.7	89.1	/	/	/	/	78.9	/	81.4	86.6	93.3
	*G*	0.20	0.20	0.12	0.18	/	/	/	/	/	/	0.33	0.23	0.23
ON vs. HC	*ACC*	81.8	81.8	77.8	89.9	78.8	60.6	78.8	77.8	82.8	81.8	81.8	84.8	80.8
	*TPR*	95.0	95.0	/	96.7	/	/	/	/	100	100	96.7	98.3	79.7
	*PPV*	79.2	79.2	/	87.9	/	/	/	/	77.9	76.9	78.4	80.8	/
	*G*	/	/	/	0.20	/	/	/	/	/	/	/	0.36	/
OFF vs. ON	*ACC*	66.7	66.7	57.5	84.2	50.0	38.3	50.8	54.2	75.0	63.3	77.3	77.5	53.3
	*TPR*	/	/	/	95.0	/	/	/	/	/	/	/	/	/
	*PPV*	/	/	/	78.1	/	/	/	/	/	/	/	/	/
	*G*	/	/	/	/	/	/	/	/	/	/	/	/	/

**Table 4:** Best-performing machine learning classifier’s characteristics and parameters.

PD vs. HC	kNNf	Accuracy	95.6%
		Goodness index	0.14
		Total misclassification cost	6
		Prediction speed	660 obs/s
		Training time	1.7149 s
		Number of neighbors	1
		Distance metric	Euclidean
		Distance weight	Equal
OFF vs. HC	DTc	Accuracy	90.9%
		Goodness index	0.12
		Total misclassification cost	9
		Prediction speed	620 obs/s
		Training time	1.3464 s
		Max number of splits	4
		Split criterion	Gini’s diversity index
ON vs. HC	kNNf	Accuracy	89.9%
		Goodness index	0.20
		Total misclassification cost	10
		Prediction speed	440 obs/s
		Training time	2.129 s
		Number of neighbors	1
		Distance metric	Euclidean
		Distance weight	Equal

**Table 5:** A comparison of this work with state-of-the-art models’ work for activity detection. PYP (Postural Yaw Perturbation), GT (Gait Task), TTHP (Toe Tapping with Heel Pin), CT (Circling Test), WLE (Walk-like events), LDA (Linear Discriminant Analysis), MLP (Multilayer Perceptron), 1D-CNN (One-dimensional Convolution Neural Network).

Author	Objective	Type of Task	ML Algorithm	Accuracy
This work	Classification of PD from HC	PYP	kNNf	96%
Aich et al. [23]	Detection of FoG	GT	SVM	88%
Caramia et al. [25]	Classification of PD from HC	GT	SVM	80%
Klucken et al. [40]	Classification of PD from HC	GT, TTHP, CT	LDA, AdaBoost, SVM	81%
Naghavi et al. [41]	Detection of FoG	GT	kNN, SVM, MLP	97%
Atri et al. [42]	Classification of PD from HC	WLE	1D-CNNs	90%

## Data Availability

The private data presented in this study are available on request from the authors.
